# *SxtA and sxtG* Gene Expression and Toxin Production in the Mediterranean *Alexandrium minutum* (Dinophyceae)

**DOI:** 10.3390/md12105258

**Published:** 2014-10-22

**Authors:** Federico Perini, Luca Galluzzi, Carmela Dell’Aversano, Emma Dello Iacovo, Luciana Tartaglione, Fabio Ricci, Martino Forino, Patrizia Ciminiello, Antonella Penna

**Affiliations:** 1 Department of Biomolecular Sciences, University of Urbino, Viale Trieste 296, Pesaro 61121, Italy; E-Mails: federico.perini@uniurb.it (F.P.); luca.galluzzi@uniurb.it (L.G.); fabio.ricci@uniurb.it (F.R.); 2 Department of Pharmacy, University of Naples Federico II, Via D. Montesano 49, Naples 80131, Italy; E-Mails: dellaver@unina.it (C.D.); emma.delloiacovo@unina.it (E.D.I.); luciana.tartaglione@unina.it (L.T.); forino@unina.it (M.F.); ciminiel@unina.it (P.C.)

**Keywords:** *Alexandrium minutum*, dinoflagellate, gene expression, genomics, HAB (harmful algal bloom), PST (paralytic shellfish toxin), saxitoxin, qPCR

## Abstract

The dinoflagellate *Alexandrium minutum* is known for the production of potent neurotoxins affecting the health of human seafood consumers via paralytic shellfish poisoning (PSP). The aim of this study was to investigate the relationship between the toxin content and the expression level of the genes involved in paralytic shellfish toxin (PST) production. The algal cultures were grown both in standard f/2 medium and in phosphorus/nitrogen limitation. In our study, LC-HRMS analyses of PST profile and content in different Mediterranean *A.* *minutum* strains confirmed that this species was able to synthesize mainly the saxitoxin analogues Gonyautoxin-1 (GTX1) and Gonyautoxin-4 (GTX4). The average cellular toxin content varied among different strains, and between growth phases, highlighting a decreasing trend from exponential to stationary phase in all culture conditions tested. The absolute quantities of intracellular *sxtA1* and *sxtG* mRNA were not correlated with the amount of intracellular toxins in the analysed *A. minutum* suggesting that the production of toxins may be regulated by post-transcriptional mechanisms and/or by the concerted actions of alternative genes belonging to the PST biosynthesis gene cluster. Therefore, it is likely that the *sxtA1* and* sxtG* gene expression could not reflect the PST accumulation in the Mediterranean *A. minutum* populations under the examined standard and nutrient limiting conditions.

## 1. Introduction

Dinoflagellates are unicellular protists that play important ecological roles in marine and freshwater habitats. Approximately 2000 species of dinoflagellates are known to date, most of which are found in marine habitats [1,2]. In the marine environment, they are important primary producers, both as free living phytoplankton and symbionts of reef forming corals or other animals [3]. Dinoflagellates also produce a wide variety of secondary metabolites including a diverse array of toxins that have a significant impact on marine ecosystems and fisheries. Almost 100 species have been identified as producers of toxic compounds that can affect humans through the trophic chain [4,5].

Nearly all the classical seafood poisoning syndromes, paralytic- (PSP), diarrhetic- (DSP), neurotoxic- (NSP), azaspiracid shellfish poisoning (AZP), and ciguatera fish poisoning (CFP), are caused by dinoflagellate toxins. One exception is represented by amnesic shellfish poisoning (ASP), which is caused by the diatomean toxin, domoic acid [6]. In addition to the classic seafood toxins, dinoflagellates may produce a range of other biologically active compounds, including cytotoxins, antibiotics and immunosuppressant compounds [7,8,9].

Saxitoxin and its analogues are potent environmental neurotoxins that can cause a severe human illness named paralytic shellfish poisoning (PSP) syndrome due to consumption of vector species, such as mussels, clams and oysters [10,11], which mainly accumulate toxins in their digestive glands. The PSP syndrome is the most widespread harmful algal bloom (HAB)-related shellfish poisoning illness [6]. It has serious impacts on human health, the marine trophic chain, marine mammals, fish, and seabirds, wild and cultured seafood, tourism and recreational activities. Dinoflagellates belonging to the genus *Alexandrium* are reported to be the main biogenetic source of PSP toxins. Indeed, these toxins are also produced by *Gymnodinium catenatum* and *Pyrodinium bahamense*, as well as by several genera of predominantly freshwater cyanobacteria [12,13]. Members of the genus *Alexandrium* are widespread globally [14]. In particular, in the Mediterranean Sea, the diversity of *Alexandrium* appears to be higher than elsewhere (12 distinct species have been identified so far from this sea) [15]. Moreover, *Alexandrium* biogeography is characterized by the distribution of toxic and non-toxic strains of the same species, or of closely related species [16]. From a chemical standpoint, PSP toxins are tetrahydropurine derivatives that include carbamoyl toxins, namely saxitoxin (STX), neosaxitoxin (NEO), gonyautoxins (GTX1 to GTX4), N-sulfocarbamoyl toxins (C1–C4, B1, and B2), and decarbamoyl toxins (dcSTX, dcNEO, and dcGTX1 to dcGTX4). A number of minor derivatives of STX have been reported both from microalgae [17] and seafood [18].

The biosynthetic pathway and genes responsible for STX synthesis have been recently identified in the cyanobacterial species *Cylindrospermopsis raciborskii*, *Anabaena circinalis*, *Aphanizomenon* spp., *Raphidiopsis brookii*, *Lyngbya wollei* and *Scytonema* sp. [19,20,21,22,23,24]. *SxtA* is the unique starting gene of the STX-synthesis in cyanobacteria. This gene has a polyketide synthase (PKS)-like structure characterized by four catalytic domains with predicted activities of a *S*-adenosyl-methionine- (SAM) dependent methyltransferase (*sxtA1*), GCN5-related *N*-acetyltransferase (*sxtA2*), acyl carrier protein (*sxtA3*) and a class II aminotransferase (*sxtA4*) [19]. In both eukaryote and prokaryote organisms, STX appears to be synthesized by similar processes; in fact, incorporation patterns of precursors (as arginine, acetate and methionine) and toxin stereochemistry are identical in both cyanobacteria and dinoflagellates [25,26,27].

In recent years, *A.* *minutum* has been studied for identifying genes and expression patterns involved in critical pathways, such as those for toxin production [28]. To identify *sxt* genes from two STX-producing *Alexandrium* species, *A. minutum* and *A. fundyense*, different molecular approaches were applied using high-throughput sequencing technology of a large number of transcripts, in *silico* transcriptome analyses, rapid amplification of cDNA ends (RACE), qPCR and conventional PCR coupled with Sanger sequencing. These multiple approaches successfully identified the genes required for STX-synthesis in dinoflagellates. This demonstrated that STX-synthesis is to be ascribed to dinoflagellates and not to co-cultured bacteria as previously hypothesized [29]. The *Alexandrium* spp. transcripts of the *sxtA* gene have the same domain structure as those from cyanobacterial homologs, but the dinoflagellate transcripts are monocistronic; they occur in multiple copies and contain typical dinoflagellate spliced-leader sequences. Furthermore, investigation of STX-producing and non-producing dinoflagellate strains of six species showed the presence of the *sxtA* gene and STX-synthesis, with exception of four *A. tamarense* strains for which *sxtA* was amplified without evidence of STX or derivatives [29].

Additionally, in the cyanobacteria, the product of polyketide synthase is the substrate for the amidinotransferase, encoded by the gene *sxtG*, which is proposed to incorporate an amidino group from an arginine molecule into the STX intermediate [30]. Recently, the characterization of the second core gene of the STX pathway in dinoflagellates, *sxtG*, was performed [31].

The aim of this study was to investigate the relationship between toxin content and expression level of the *sxtA1* and *sxtG* genes in the Mediterranean *A. minutum*.

It is known that the production of toxins in some *Alexandrium* spp. can be influenced by nutritional conditions. In particular, low levels of nitrate cause the decrease of toxicity, while low levels of phosphorus increase it [32,33,34,35]. Therefore, we conducted experiments also in conditions of nutrient depletion to check how these conditions could affect the toxins produced in *A. minutum* isolated from the Mediterranean Sea and if these nutritional factors could affect the regulation of *sxtA* and *sxtG* gene expression.

## 2. Results and Discussion

### 2.1. Toxin Content in Standard Condition

**Figure 1:**
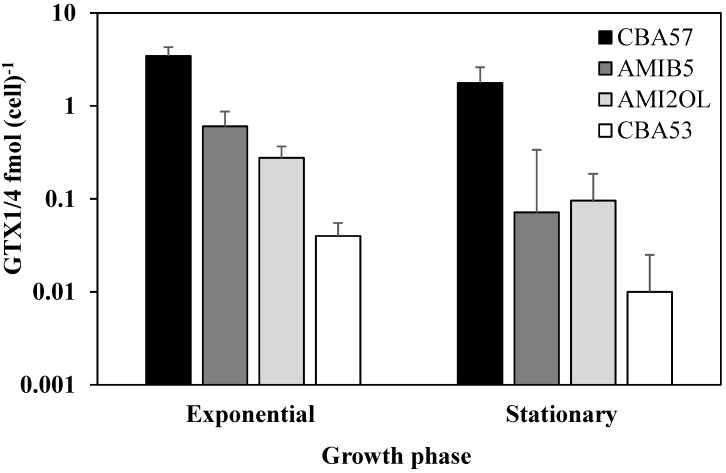
Intracellular GTX1/4 content in the Mediterranean *A. minutum* strains during the exponential and stationary growth phases (means ± SD, *n* = 3).

Liquid chromatography coupled with high resolution mass spectrometry (LC-HRMS) was used to check the presence of all the major STX derivatives. The HILIC-MS/MS method for PSP toxins developed by Dell’Aversano* et al.*, on a triple quadrupole MS [36] was slightly modified to make it suitable to HRMS detection. All the analyzed strains were found to produce only GTX1 and GTX4, which differ from each other only in stereochemistry at one chiral center. They were produced at higher levels in the exponential phase than in the stationary phase. The intracellular content of toxins varied among strains in the two growth phases (*p* < 0.05). The CBA57 strain turned out to be the most productive one (GTX1 + GTX4 = 3.45 fmol cell^−1^ in the exponential phase, and GTX1 + GTX4 = 1.77 fmol cell^−1^ in the stationary phase), while the CBA53 strain was the lowest productive one (GTX1 = 0.04 fmol cell^−1 ^in the exponential phase, and GTX1 = 0.01 fmol cell^−1^ in the stationary phase). In the AMIB5 and CBA57 strains, the toxin content significantly declined during cell growth from the exponential to the stationary phases (*p* < 0.05). However, the decrease of GTXs content from the exponential to the stationary phase was also observed in the other two strains, although it was not statistically significant (Figure 1). In almost all the analyzed samples, the GTX4 concentration was higher than that of GTX1, with the exception of the CBA53 strain that produces only GTX1. In particular, in the exponential phase the content of GTX4 was three times higher than GTX1 in the AMIB5 and AMI2OL strains, and 4.5 times in the CBA57 strain. In the stationary phase, only the GTX4 declined significantly in the CBA57 strain and the same trend was observed in the other strains. In the *Alexandrium* strains, the composition of toxins is related to the phenotypic trait, but the amounts are variable among strains. Members of the *A. minutum* group (as well as *A. ibericum, A. lusitanicum, A. angustitabulatum*) produce primarily gonyautoxins, such as GTX1 and GTX4 [14,37]. Average cellular toxin content of toxigenic *Alexandrium* isolates varies considerably (up to an order of magnitude) among different growth phases and environmental regimes in batch cultures [38,39]. Moreover, the toxin content of *Alexandrium* strains isolated from the same geographical area can be extremely variable (from undetectable levels to >100 fmol cell^−1^ of toxins) [36]. The GTXs toxin content and composition of the Mediterranean *A. minutum* strains used in this study was in agreement with the previous observations [40,41].

### 2.2. The *sxtA1* and *sxtG* Gene Expression in Standard Condition

The use of endogenous housekeeping genes (actin or 5.8S rRNA) for relative quantification analyses did not produce reliable results due to the high expression variability of these genes between the two growth phases. Therefore, an absolute quantification approach was adopted using standard curves constructed with scalar dilutions of the *sxtA1* and *sxtG* PCR products. A fixed amount of human RNA was spiked in each reverse transcription reaction and human β2M gene transcript was amplified in each cDNA sample to control the efficiency of reverse transcription and to indicate possible inhibitory effects during the synthesis of the cDNA. Moreover, the β2M was also used as an exogenous housekeeping gene for relative quantification. Using this exogenous reference, the relative quantification data showed the same trend obtained with absolute quantification (data not shown). The *sxtA1* and* sxtG* standard curves showed efficiency of 98% and 99% and good linear regression (R^2^ = 0.99) (Supplementary Figure S1).

**Figure 2:**
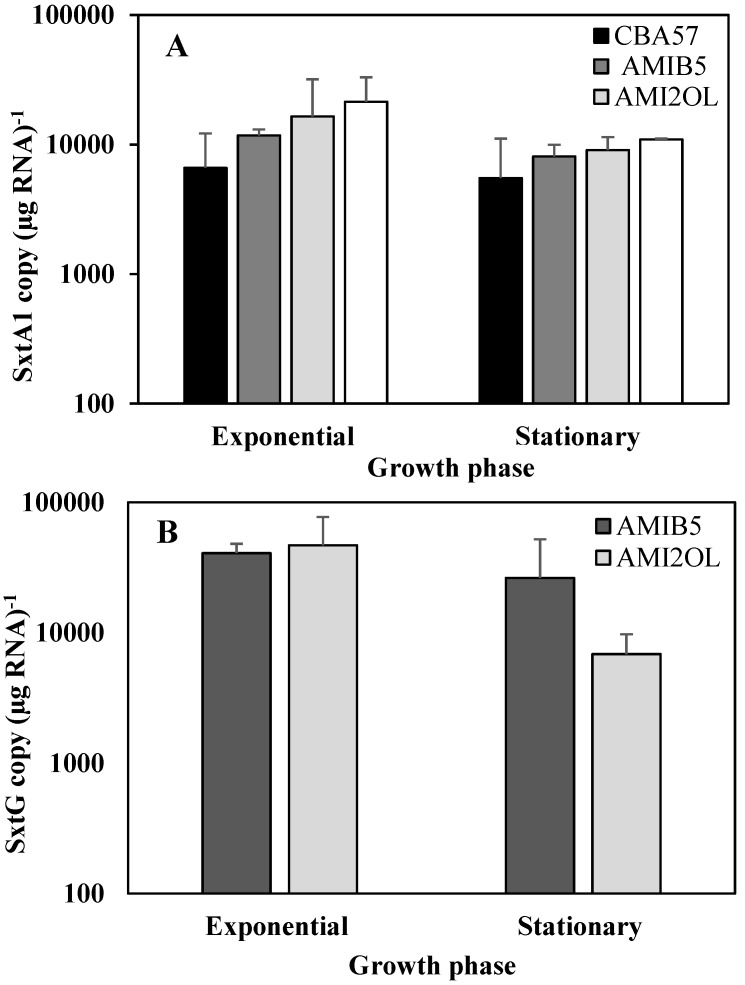
The *sxtA1* and *sxtG* gene expression in standard nutrient condition. Absolute quantification of *sxtA1* (**A**) and *sxtG* mRNA (**B**) during the exponential and stationary phases of growth (means ± SD, *n* = 3). The *sxtG* expression was determined only in two strains of *A. minutum* (AMI2OL and AMIB5), the only ones for which the PCR amplification was confirmed with our specific primers using genomic DNA.

The number of *sxtA1* and* sxtG* transcripts was calculated by plotting the Ct of each sample on the standard curve. The data were normalized per μg of total RNA (Figure 2).

In the exponential phase, the CBA53 strain showed a content of the *sxtA1* mRNA significantly higher than that of other strains (*p* < 0.05). Although a reduction of *sxtA1* expression during the stationary phase compared to the exponential phase was evident for all strains; a statistically significant difference was observed only for the strain CBA53 (*p* < 0.05) (Figure 2A).

Curiously, the *sxtG* was not detected in the most toxic CBA57 strain, or in the CBA53 strain. This finding was also confirmed by experiments with internal positive control (see 3.4 in Experimental Section) demonstrating that the negative results was not an artifact due to inhibition of the PCR reactions. Therefore, the *sxtG* gene expression analyses were performed on AMI2OL and AMIB5 strains only. No significant differences of *sxtG* transcript abundance were observed between the two strains in both growth phases, but significant difference was found between the two growth phases in the AMI2OL strain (*p* < 0.05) (Figure 2B). Moreover, the *sxtG* expression did not correlate with *sxtA1* gene expression in strains AMI2OL and AMIB5.

Unlike *sxtA* gene, the presence of *sxtG* gene is not exclusively specific of the *Alexandrium* species reported to produce saxitoxins [31]. In fact, it was observed that the *sxtG* amidinotransferase was present and transcribed in *Alexandrium* species where the *sxtA* gene and STX synthesis have not been identified [13]. Therefore, this gene could also be involved in other biochemical pathways or these *Alexandrium* spp. could have lost the capacity of STX synthesis [31]. In this study, the absence of *sxtG* gene in CBA57 and CBA53 strains of *A. minutum* could be related to the presence of a homolog of amidinotransferase that was not amplified by our primers. In fact, a second dinoflagellate amidinotransferase that groups more distantly to *sxtG* with homologous actinobacterial and cyanobacterial cylindrospermopsin *aoaA* and *cyrA* sequences has been identified suggesting that multiple amidinotransferases have been acquired by horizontal gene transfer (HGT) in parallel or separate events during *Alexandrium* evolution [31].

Each amount of *sxtA1* or *sxtG* mRNA measured in the two growth phases was compared to the amount of toxins (GTX1/4) produced. No significant correlations were found between amount of mRNAs and intracellular toxin content in all the strains. Unexpectedly, the less toxic CBA53 strain showed the highest level of *sxtA1* gene expression. Moreover, the mRNA transcripts and toxins content were compared along the different growth phases. In all the strains, even if no significant correlation was found, the mRNA transcripts and toxins showed the highest and lowest contents in the exponential and stationary phases, respectively.

The absence of correlation between the gene expression and toxin content could be due to the fact that the PSP toxin biosynthesis enzymes are long-living enzymes with a slow turn-over and may be regulated by post-translational mechanisms [30]. Moreover, in the cyanobacteria* C. raciborskii* T3, the saxitoxin biosynthetic pathway is encoded by a gene cluster of more than 35 kb, and comparative sequence analysis assigns 30 catalytic functions to 26 proteins [19]. A cluster of 14 genes, defined as “core” genes (*sxtA–sxtI*,* sxtP–sxtS* and* sxtU*) is common between the STX-pathways of several cyanobacterial genera [11,42]. Eight of these genes (*sxtA*,* sxtB*,* sxtD*,* sxtG*,* sxtH/T*,* sxtI*, *sxtS* and *sxtU*) seem to be directly implicated in STX-synthesis [19]. The STX biosynthesis pathway appears conserved between cyanobacteria and dinoflagellates: it involves arginine, SAM synthetase and acetate, with the addition of the methyl group of SAM into the final molecule [43]. It is likely that in *A. minutum*, saxitoxin and its derivatives are the result of the synergistic action of several enzymes homologous to those of cyanobacteria [13,29]. Of the 14 “core” STX genes, 10 dinoflagellate homologues, or candidate genes, are presently identified (*sxtA*,* sxtB*,* sxtD*,* sxtF–I*,* sxtQ*,* sxtS* and *sxtU*) [29,44]. However, sequence conservation might be so low that reliable homologue identification is impossible or, if several homologues are indeed missing in the dinoflagellates, alternative dinoflagellate genes could have substituted their functions in the SXT pathway. Alternatively, the STX biosynthetic pathway could have evolved independently in cyanobacteria and dinoflagellates [44]. Moreover, dinoflagellates have large genomes, a considerable number of unknown genes and a high frequency of repeats making genomic studies very hard [27].

Stüken* et al.* (2011) characterized the *sxtA* gene showing a comparable domain structure to its cyanobacterial homologue [29]. *SxtA* encodes a polyketide synthase, the first enzyme in the metabolic pathway, but it is not clear which other genes are involved and the extent of their activity. This fact is to be considered when the amount of intracellular toxins needs to be correlated to gene expression. Also, the subsequent characterization of *sxtG*, the second “core” gene in the STX pathway [31], may indicate a massive transfer of toxin-related genes from bacteria to dinoflagellates [45]. However, in contrast to cyanobacteria, most of the genes involved in STX-synthesis in dinoflagellates have remained elusive.

### 2.3. The sxtA1 Gene Expression and Toxin Content under Phosphorus Limitation

**Figure 3:**
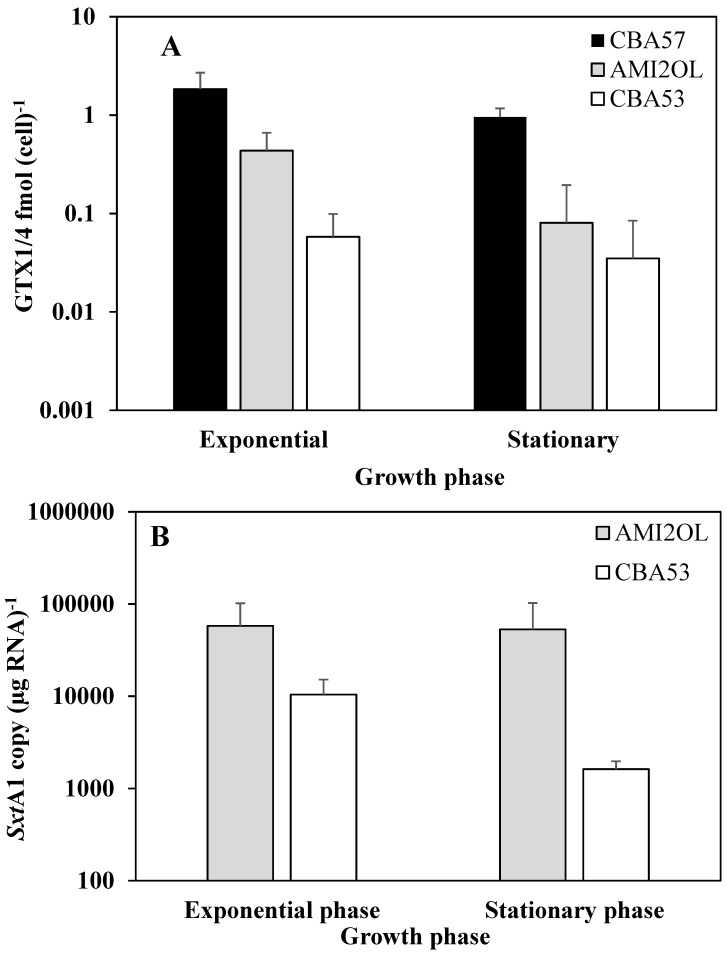
Intracellular toxin content in the Mediterranean *A. minutum* strains during the exponential and stationary growth phases (means ± SD, *n* = 3) (**A**) and *sxtA1* gene expression as mRNA copy per µg RNA^−1^ (**B**) under phosphorus limitation (means ± SD, *n* = 3). The *sxtA1* gene expression was undetectable in the CBA57; thus, *sxtA1* copy (µg RNA^−1^) values were omitted.

Strains of CBA57, AMI2OL and CBA53 were grown in phosphorus limitation as described in the Experimental Section. Under this condition, all strains were characterized by a short exponential phase; therefore, all withdrawals were made at the fifth or sixth day (exponential phase) and at the 12th day (stationary phase) (Supplementary Figure S2). The concentrations of dissolved inorganic phosphorus varied from 0.16 ± 0.1 μM (day 1) to 0.13 ± 0.04 μM (day 12) (Supplementary Table S1). The daily decrease in dissolved phosphorus is extremely low compared to the variation of the standard condition. This could be due to the fact that the algal biomass that develops in these limiting conditions is much lower compared to the standard conditions. In the phosphorus limitation, the toxin content tended to decrease from exponential to stationary phase confirming the same trend of the standard conditions (Figure 3A). However, this decrease was not significant with the exception of AMI2OL strain (*p* < 0.05).

Under phosphorus limitation, the *sxtA1* gene expression decreased significantly only in the CBA53 strain between the exponential and stationary phase. No significant variation of *sxtA1* gene expression was observed in the AMI2OL strain (Figure 3B). Furthermore, in CBA57 strain, which expressed the lowest *sxtA1* copy number in the standard condition, the expression of *sxtA1* gene was undetectable in contrast to the higher GTX1/4 content respect to the other *A. minutum* strains.

The toxin content and mRNA levels at each stage of growth for all strains were compared with the values obtained in the standard growth condition. No significant differences of GTX1/4 content were observed between standard and phosphorous depletion for strains CBA53 and AMI2OL, while the CBA57 strain showed a significant toxin content decrease (*p* < 0.05) in phosphorous limitation both in exponential and stationary phases. It is noteworthy that in phosphorous depletion, GTX2 and GTX3 were detected in trace amounts in the CBA57 strain only (data not shown).

As for the *sxtA1* gene expression, a significant decrease of the mRNAs was found in CBA53 strain compared to the standard nutritional condition. In particular, in the CBA57 strain, which expressed the lowest *sxtA1* copy number under the standard condition, the expression of *sxtA1* gene was undetectable along with an evident decreasing of toxins. Instead, in the AMI2OL strain, the *sxtA1* gene expression did not vary much in both the exponential and stationary phases compared to the standard growth conditions. However, the Spearman’s correlation analysis confirmed the independence of gene expression and GTX1/4 intracellular content for each strain.

Some authors reported accumulation of intracellular PST in phosphorus limiting conditions [33,34,35,46,47]. However, in these studies, almost all the *A. minutum* strains were not from Mediterranean areas with the exception of the *A. minutum* A5 strain. This strain in the phosphorous limitation did not show significant effect on toxin production [47]. In that study, a rapid and substantial increase in PST levels was observed in *A. minutum* strains in the presence of waterborne grazers, suggesting that secondary metabolism of dinoflagellates is not only dependent on resource availability, but also on the predation pressure. In fact, it was also suggested that PSTs may be produced in phosphate-limited conditions in order to redirect the grazing pressure toward alternative non-toxic competitors [32]. In the nutritional conditions of our study, the strains were not subjected to pressure from predators. Furthermore, since phosphorus is involved in the energetic metabolism and in the regulation of intracellular functions, its deficiency in the medium should negatively affect the nucleotide synthesis, as well as the energy reserves of the cell. As a consequence, it is logical to suppose that cell energy is likely employed for the maintenance of basic and essential cellular functions [35]. Hence, the activation of energetically costly pathways, such as that used for saxitoxin synthesis, would not be activated if not necessary. This could explain the down-regulation of the *sxtA1* gene in our Mediterranean *A. minutum* strains during phosphorus depletion treatment. The different behaviour of the *A. minutum* AMI2OL could be due to the biological/genetic variability of the strain [48], and this has to be further investigated by using a higher number of Mediterranean* A. minutum* strains.

### 2.4. The sxtA1 Gene Expression and Toxin Content under Nitrogen Limitation 

The *sxtA1* gene expression under nitrogen limitation was tested on the CBA57 and AMIB5 strains. As observed for *A. minutum* strains grown in phosphorus limitation, the growth was characterized by a short exponential phase; therefore, sample withdrawals were made at the fifth or sixth day (exponential phase) and the 12th day (stationary phase) (Supplementary Figure S2). The concentration of total dissolved nitrogen varied from 77.5 ± 6.5 μM at the inoculation time to 0.73 ± 0.1 μM at day 12 (Supplementary Table S1).

**Figure 4:**
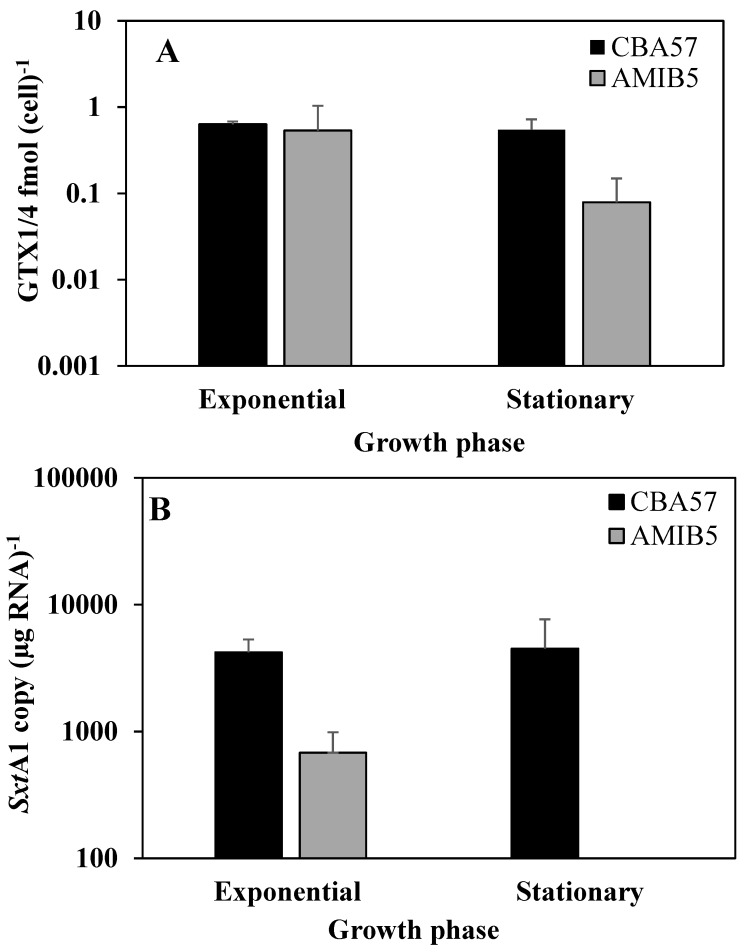
Intracellular toxin content in the Mediterranean *A. minutum* strains during the exponential and stationary growth phases (means ± SD, *n* = 3) (**A**) and *sxtA1* gene expression as mRNA copy per µg RNA^−1^ (**B**) under nitrogen limitation (means ± SD, *n* = 3). In the *A. minutum* AMIB5 strain, the *sxtA1* expression was not detected in the stationary phase.

Both strains produced similar toxin amounts in exponential and stationary phases with no significant differences (Figure 4A). For the CBA57 strain, a significant reduction in toxin intracellular content was found in both exponential and stationary phases with respect to the standard conditions (*p* < 0.05), while in the AMIB5 strain this difference was not significant. In the CBA57 strain, the *sxtA1* gene was constantly expressed under nitrogen limitation, either in the exponential and stationary phase (Figure 4B). In the AMIB5 strain, the *sxtA1* expression was strongly down regulated in the stationary phase. With respect to the standard conditions, expression of *sxtA1* in strain CBA57 did not change significantly, while it was strongly down regulated in the AMIB5 strain (*p* < 0.05). 

The effect of nitrogen limitation in toxin production by the CBA57 strain was consistent with previous studies [35,47]. This effect is reasonable because toxins are nitrogen-rich molecules, which might be synthesized as a by-product of amino acids or nitrogen excess. In fact, in nitrogen limitation conditions, the intra-cellular pools of nitrogen would mainly be allocated to the production of nucleotides and amino acids in order to maintain basic and essential cellular functions, while the activation of nitrogen demanding metabolic pathways, such as PST biosynthesis, would not be favoured [46]. On the other hand, the fact that intracellular content of GTX1/4 did not change significantly with respect to the standard conditions in AMIB5 may be due to low amounts of toxin production. In this case, the amount of nitrogen in the medium may be sufficient to maintain the toxin levels observed in the standard conditions.

## 3. Experimental Section

### 3.1. Strain Cultures 

*Alexandrium minutum* monoclonal strains used in this study were isolated from surface sea water in the Mediterranean Sea. Strains CBA53 and CBA57 were from Adriatic Sea (43°46'19''N, 13°09'24''E) and AMIB5 and AMI2OL were from Ionian Sea (37°03'04''N, 15°17'02''E) and Tyrrhenian Sea (40°57'11''N, 09°39'04''E), respectively. The sampled localities are mesotrophic areas. The localities of the Ionian Sea and Tyrrhenian Sea are closed areas characterized by freshwater inputs; the coastal north-western Adriatic Sea is strongly influenced by the Po river input determining the trophic conditions of seawaters [49,50].

Cultures were maintained in 2 L bottles, using standard conditions of f/2 medium minus silicate [51], at 21.5 ± 1 °C, light irradiance of 100 µmol m^−2^s^−1^ for 12:12 h light-dark cycles. In addition to these standard conditions, CBA53 and AMI2OL strains were also cultured in phosphorus starvation, the strain AMIB5 in nitrogen starvation and strain CBA57 in both phosphorous and nitrogen starvation. The concentrations of nutrient and growth conditions are summarized in Table S1. Every two days each culture was assessed for cell concentrations and growth rate by Utermöhl method [52] using an inverted microscope (Axiovert 40 CFL, Zeiss, Göttingen, Germany).

The stock cultures to be further inoculated in batch cultures were grown with antibiotics (50 μg mL^−1^ ampicillin, 33 μg mL^−1^ gentamicin, 10 μg mL^−1^ ciprofloxacin, 1.13 μg mL^−1^ chloramphenicol and 0.025 μg mL^−1^ streptomycin sulphate) using sterile handling techniques to minimize bacterial influence, as described in [40]. The antibiotic treatment was stopped at inoculation of the cultures while always maintaining aseptic handling techniques to avoid any bias introduced by this handling. The cells were analysed in the exponential (growth rate 0.21 ± 0.06 µ day^−1^) and stationary (growth rate 0.03 ± 0.01 µ day^−1^) growth phases (Supplementary Figure S3). The duration of culture experiments was between 12 and 31 days, depending on the growth rates. Cells were harvested by filtration using 3 μm pore-sized filters, rinsed with sterilised seawater and centrifuged 10 min at 1200× *g*. The pellets were immediately stored at −80 °C until total RNA extraction or PSP toxin analysis. The eluates were stored at −20 °C and used for the nutrient analyses.

### 3.2. Toxin Analysis

#### 3.2.1. Chemicals

All organic solvents, glacial acetic acid, formic acid, and ammonium formate (Laboratory grade) were from Sigma-Aldrich Corporation (St. Louis, MO, USA). Standard solutions of PSP toxins were provided by the NRC Certified Reference Materials Program (Institute for Marine Biosciences, Halifax, NS, Canada). Nine concentration levels were prepared from a mixture of certified standards by serial dilutions in water 0.1 M acetic acid, starting with the following concentrations: B1 (1370 ng/mL), C1 (1500 ng/mL), C2 (450 ng/mL), STX (340 ng/mL), GTX2 (1250 ng/mL), GTX3 (480 ng/mL), NEO (290 ng/mL), GTX1 (690 ng/mL), GTX4 (230 ng/mL), dcSTX (590 ng/mL), dcGTX2 (1140 ng/mL), dcGTX3 (260 ng/mL), and dcNEO (280 ng/mL). A mixture containing the above toxins was also used to optimize HRMS parameters.

#### 3.2.2. Pellet Extraction

Pellet of the four different strains of *A. minutum* (CBA53, CBA57, AMIB5 and AMI2OL) were separately extracted. Each pellet was added of 1 mL aqueous 0.1 M acetic acid and sonicate for 10 min. in pulse mode, while cooling continuously in an ice bath. The mixture was centrifuged at 4835× *g* for 10 min and the supernatant was decanted so as to obtain 1 mL extract that was directly analysed by LC-HRMS.

#### 3.2.3. LC-HRMS

All LC-HRMS analyses were performed on an Agilent 1100 LC binary system (Palo Alto, CA, USA) which included a solvent reservoir, in-line degasser, binary pump and refrigerated autosampler coupled to a hybrid linear ion trap LTQ Orbitrap XL™ Fourier transform mass spectrometer (FTMS), equipped with an ESI ION MAX™ source (Thermo-Fisher, San Josè, CA, USA). 

A 5 μm TSK-gel Amide-80^®^ column (250 × 2.0 mm i.d.) (Tosoh Bioscience LLC, 156 Keystone Drive, Montgomeryville, PA, USA) was eluted isocratically at 0.2 mL/min with 65% B. Eluent A was water and B was a 95% acetonitrile/water solution, both eluents contained 2.0 mM ammonium formate and 3.6 mM formic acid (pH 3.5) [36]. The injection volume was 5 µL.

LC-HRMS analyses were performed in the positive ion mode, in collision induced dissociation (CID) MS^2^ experiments, by using the following source settings: spray voltage = 4.2 kV, capillary temperature = 440 °C, capillary voltage = 29 V, sheath gas = 35 and auxiliary gas= 10 (arbitrary units), tube lens voltage= 70 V. In all experiments, a 30,000 resolving power was used. Full scan (FS) spectra were collected in the mass range *m*/*z* 200–500, MS/MS spectra were acquired by using the parameters reported in able 1, with an activation Q of 0.250, and an activation time of 30 ms. Extracted ion chromatograms (XIC) were obtained from MS/MS spectra by selecting fragment ions reported in Table 1 and used for quantification* versus* PSP toxin standards.

**Table 1:** Precursor ion, formula, mass range, and collision energy (%), used in the chemical analyses, and limit of detection (LOD) and quantification (LOQ) measured.

Toxin	Precursor Ion (*m/z*)	Formula	Product Ion (*m/z*)	Collision Energy (CE) %	LOD (ng/mL)	LOQ (ng/mL)
GTX1/4	332.1	[M + H − SO_3_]^+^	314.1204 253.1041	20	5/30	11/56
STX	300.1	[M + H]^+^	282.1311 221.1143 204.0877	22	21	40
B1	300.1	[M + H − SO_3_]^+^	282.1311 221.1143 204.0877	22	43	86
dcSTX	257.1	[M + H]^+^	239.1255 222.0984 180.0765	24	37	70
GTX2/3	316.1	[M + H − SO_3_]^+^	298.1254 220.0824	21	40/30	78/60
C1/2	316.1	[M + H − 2SO_3_]^+^	298.1254	21	23/28	47/60
NEO	316.1	[M + H]^+^	298.1254 220.0824	21	9	18
dcGTX2/3	273.1	[M + H − SO_3_]^+^	255.1201 238.0933	25	71/60	140/130
dcNEO	273.1	[M + H]^+^	255.1201	25	70	140

Calculation of elemental formulae was performed by using the mono-isotopic ion peak of each ion cluster. A mass tolerance of 5 ppm was used and the isotopic pattern of each ion cluster was considered. For each PSP toxin, the limit of detection (LOD) was measured and corresponded to the lowest concentration level that can be determined at 3 < S/N ratio < 10. Limit of quantification (LOQ) was measured and corresponded to the lowest concentration level that can be determined at S/N ratio >10 (Table 1).

#### 3.2.4. Matrix Effect

An *A. minutum* sample, containing only GTX1/4, was spiked with a pure GTX2/3 standard to obtain a concentration level of 63 ng/mL. Matrix effect was calculated by comparing the peak area of the matrix matched (MM) standard with that of a matrix free (MF) GTX2/3 standard. 

The ion suppression or enhancement effect was assessed as: 100 – (peak area of MM standard/peak area of MF standard) × 100.

### 3.3. RNA Extraction and Reverse-Transcription

Each strain was analysed for absolute and relative quantification of *sxtA* and *sxtG* mRNAs content. RNA was extracted from 3.0 × 10^6^ cells at different growth phases using TRIzol Reagent (Ambion, Life Technologies, Carlsbad, CA, USA) following manufacturer’s instructions with few modifications: cell lysis was performed at 60 ± 1 °C for 10 min in a water bath, and there was a 10 min shaking step with 0.5 mm zirconia-silica beads (400 mg) contained in the sample tube. The resulting RNA pellet was dissolved in 100 μL RNAse-free water and purified with the RNeasy mini kit (Qiagen, Hilden, Germany) including on-column DNase digestion with the RNase-Free DNase Set (Qiagen, Hilden, Germany). Finally, RNA was eluted with 40 μL RNase-free water. The concentration, integrity and purity of RNA was tested with a PharmaSpec UV-1700 spectrophotometer (Shimadzu, Kyoto, Japan), measuring absorbance at 260, 280 and 230 nm, and with electrophoresis analysis in an agarose gel. Only samples with intact RNA were taken into account for reverse-transcription and qPCR. The purified RNA (900 ng) was spiked with 100 ng of human RNA derived from human MCF7 cells, to be used as an exogenous reverse transcription control. The cDNA was prepared using SuperScript^®^ III First-Strand Synthesis SuperMix for qRT-PCR (Invitrogen, Life Technologies, Carlsbad, CA, USA).

### 3.4. Primer Design and qPCR Conditions

The primers were designed using Primer Express 2.0 software (Applied Biosystems, Life Technologies, Carlsbad, CA, USA). The sequences used for designing primers specific for genes of interest were: a long isoform precursor mRNA of *sxtA* (GenBank Accession number: JF343268) and mRNA *sxtG* (GenBank Accession number: JX995121) both from *Alexandrium minutum* CCMP113. These primers and condition were used for amplification of both cDNA and genomic DNA for assessing the presence of target sequence in the strain genomes. Experiments that gave a negative result were repeated by including within the same reaction 100 copies of purified PCR product as a positive control in order to verify the absence of inhibition.

The actin and 5.8S RNA genes were initially considered as housekeeping genes (HKG)*.* However, due to their significant differences in expression observed at the different growth phases, the human β2M gene was used as a control for relative quantification of *sxtA* and* sxtG* expression, and to check the reproducibility and efficiency of reverse transcription. The actin primers were designed using the *A. minutum* actin gene as reference (JN402307). The specificity of all primers was examined *in silico* using BLAST. The primers specific for 5.8S RNA were 5.8S3′ and 5.8S5′ [53]. The primers β2M f and β2M r specific for human β2-microglobulin were from [54].

The primer sequences and concentrations used in each qPCR reaction are shown in Table 2. The qPCR protocols were performed on a StepOne Real-time PCR system (Applied Biosystems, LifeTechnologies, Carlsbad, CA, USA) and have been optimized in order to obtain reaction efficiencies close to 100%. Reactions were run in 25 µL volumes with Hot-Rescue Real-Time PCR Kit-SG Mix (1×) containing Sybr Green (Diatheva, Fano, Italy). All qPCR amplification protocols started with a 10 min activation step at 95 °C, followed by 40 cycles including 15 s at 95 °C, and 1 min at 60 °C for annealing and extension. The qPCRs were followed by a dissociation protocol from 60 °C–95 °C and melting curve analysis. All samples were run in three biological replicates and each of those were run with two technical replicates.

**Table 2:** Primers designed in this study or previously developed including optimized final concentrations for the real-time PCR assay.

Primers	Sequences 5′→3′	Concentration
sxtA1 alex. F sxtA1 alex. R	GCAGCGATGCTACTCCTACTACGT Tcgaagakgatgckgtggtacct	600 nM 600 nM
sxtG F sxtG R	Ccgggccgtgaaggat tgtggctcgtcgatttcga	600 nM 600 nM
Act a.min upp. Act a.min low.	Agattgtgcgcgatgtcaagg cgccgtgatgatgattccctc	400 nM 400 nM
5.8S 3′ 5.8S 5′	[53]	400 nM 400 nM
β2M F β2M R	[54]	200 nM 200 nM

The standard curves were constructed from a six point ten-fold dilution series of purified *sxtA1* and *sxtG* PCR products (from 2 to 1.0 × 10^6^ copies) generated from DNA of *A. minutum* AMI2OL. The PCR product was purified with the MinElute Gel Extraction Kit (Qiagen, Hilden, Germany) and quantified with a Qubit (Invitrogen, LifeTechnologies, Carlsbad, CA, USA). The amplification efficiency of the qPCR assays was estimated from standard curves using the StepOne software version 2.3 (Applied Biosystems, LifeTechnologies, Carlsbad, CA, USA).

### 3.5. Nutrient Analyses

Chemical analyses of dissolved inorganic nutrients (N-NO_3_, N-NO_2_, N-NH_4_ and P-PO_4_) in culture supernatants filtered on 0.45 µm nitrocellulose filters (Millipore, Temecula, CA, USA) were performed following the method of [55] using a Shimadzu spectrophotometer (mod. UV-1700). Dissolved inorganic nitrogen (DIN) was expressed as NO^−^_3_ + NO^−^_2_ + NH^+^_4_ and dissolved inorganic phosphate (DIP) was expressed as P-PO_4._

### 3.6. Statistical Analyses

Shapiro–Wilk test was used to check the lack of normality of data. Therefore, the nonparametric tests Mann–Whitney and Kruskal–Wallis were used for the comparison of median among strains and growth phases, for gene expression data and the determination of toxin intracellular contents. The Spearman’s test was used for the correlation between levels of gene expression and intracellular content of toxins. All tests were obtained with PAST ver. 2.09 [56] with a *p* < 0.05 determining significance.

## 4. Conclusions

This is the first study on *sxtA1* and *stxG* gene expression and their correlation with toxin content in Mediterranean *A. minutum*. The expression levels and intracellular toxin accumulation were studied in *A. minutum* strains grown in enriched medium and nutrient limitation. In standard medium conditions, *A. minutum* produced exclusively GTX1/4 and the toxin production decreased from exponential to the stationary phase. Despite both the *sxtA1* gene expression and intracellular toxin content showing a reduction in trend from exponential to stationary phase, this correlation was not significant. The *sxtG* gene was detected in only two strains. The gene expression followed the same trend of the *sxtA1*, but the absolute mRNA quantity was not correlated with either toxins or with *sxtA1*. Under phosphorus or nitrogen limitation, the toxin content displayed a significant reduction in the *A. minutum* CBA57 only. Also in these nutrient depleted conditions, the correlation between gene expression and toxin was not significant.

Hence, the monitoring of expression level of *sxtA1* and *sxtG* did not appear sufficient to predict toxicity in Mediterranean *A. minutum*. It would be necessary to increase knowledge regarding the expression and function of other genes involved in the PST biosynthesis pathway to improve the ecological interpretation of toxin production, as well as to provide the possibility of using new molecular markers for the monitoring of toxin presence in seawater and accumulation in farmed shellfish.

## Supplementary Files

Supplementary File 1
