# Supplementary material for: SxtA and sxtG Gene Expression and Toxin Production in the Mediterranean Alexandrium minutum (Dinophyceae)
Source: Mar Drugs. 2014 Oct 22;12(10):5258–76. doi: 10.3390/md12105258 (PMC4210898; doi:10.3390/md12105258)
Supplement: Supplementary File 1 [file marinedrugs-12-05258-s001.pdf]

Supplementary Information

Figure S1. Standard curves constructed with PCR products of *sxtA* (**A**) and *sxtG* genes (**B**). The curves were the mean of the three different experiments.

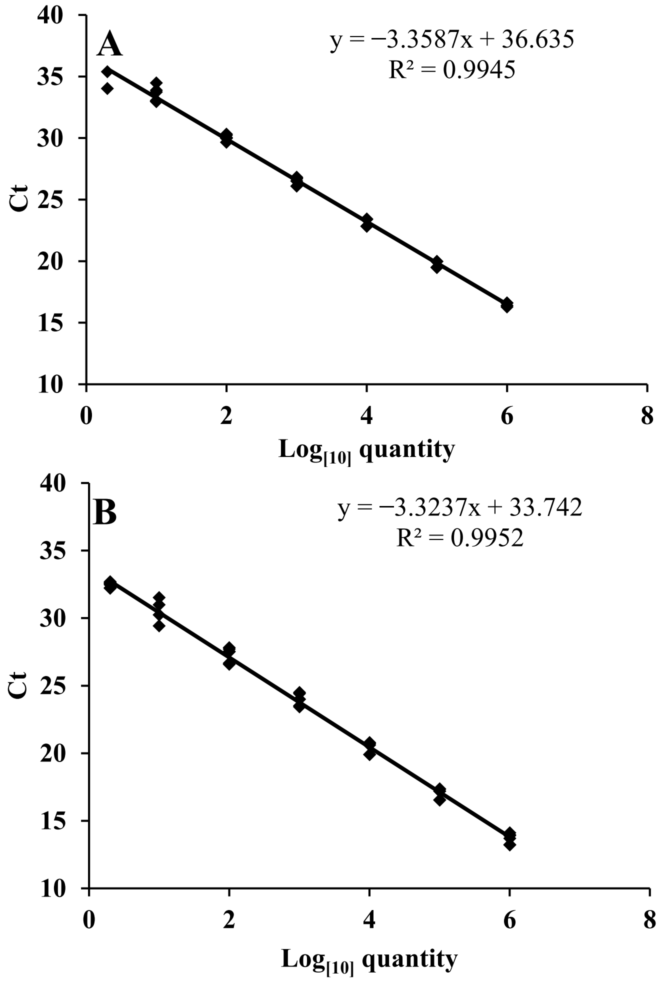

Figure S2. Cell densities (cells ml^{-1}) of the representative *Alexandrium minutum* CBA57 strain under phosphorous and nitrogen limiting conditions (means ± SD, *n* = 3).

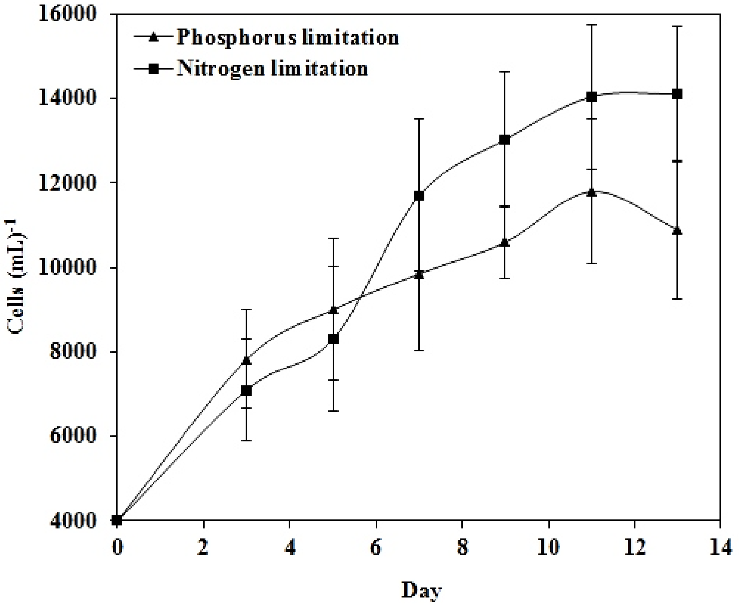

Figure S3. Growth curves of the *Alexandrium minutum* strains in standard nutritional conditions. The curves were the mean of the three biological replicates (means ± SD, $n = 3$).

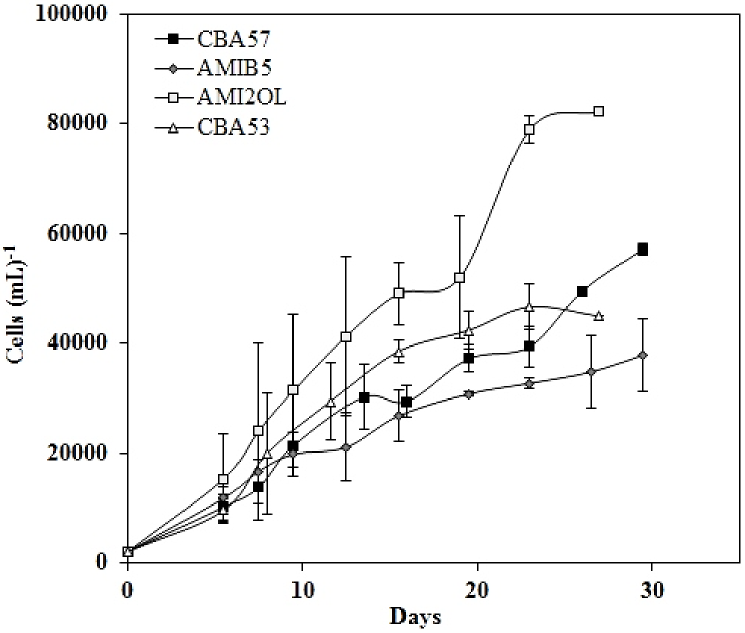

Table S1. Concentration of dissolved inorganic nitrogen (DIN) and dissolved inorganic phosphorus (DIP) in the different growth media conditions and growth phase. T_0 correspond to the time of inoculation (day 1).

Nutrients (µM)	Standard Condition			Phosphorous Limitation			Nitrogen Limitation		
	T_0	Exponential Phase	Stationary Phase	T_0	Exponential Phase	Stationary Phase	T_0	Exponential Phase	Stationary phase
**DIN**	951.6 ± 28.3	678 ± 58.2	142.6 ± 40.4	916.9 ± 24.2	899.2 ± 89.1	764.2 ± 67	77.5 ± 6.5	21.5 ± 2.1	0.73 ± 0.1
**DIP**	36.3 ± 1.0	14.8 ± 3.9	0.45 ± 0.1	0.16 ± 0.1	0.16 ± 0.05	0.13 ± 0.04	36.7 ± 0.6	21.1 ± 2.1	9.63 ± 0.9

© 2014 by the authors; licensee MDPI, Basel, Switzerland. This article is an open access article distributed under the terms and conditions of the Creative Commons Attribution license (http://creativecommons.org/licenses/by/3.0/).
